# Relationship between relative skeletal muscle mass and nonalcoholic fatty liver disease: a systematic review and meta-analysis

**DOI:** 10.1007/s12072-019-09964-1

**Published:** 2019-07-09

**Authors:** Changzhou Cai, Xin Song, Yishu Chen, Xueyang Chen, Chaohui Yu

**Affiliations:** Department of Gastroenterogy, The First Affiliated Hospital of Zhejiang University School of Medicine, Hangzhou, 310003 China

**Keywords:** Nonalcoholic fatty liver disease, Steatohepatitis, Liver fibrosis, Sarcopenia, Skeletal muscle mass

## Abstract

**Background and Aim:**

Nonalcoholic fatty liver disease (NAFLD) has gradually become one of the most common chronic liver diseases in the world. More and more evidence shows that low skeletal muscle mass index (SMI) may play a role in the development of NAFLD. Our aim was to quantify the association between SMI, sarcopenia and the presence and severity of NAFLD.

**Methods:**

We systematically searched English relevant studies from PubMed, Embase, the Web of Science and the Cochrane Library updated to December 20th, 2018. Studies in which SMI was compared between NAFLD cases and controls were included. So were studies concerning the odds ratio (OR) of NAFLD, non-alcoholic steatohepatitis (NASH) and significant fibrosis in sarcopenia patients. Pooled weighted mean differences and ORs were calculated.

**Results:**

Of the 1331 retrieved studies, 19 articles were included. SMI level in NAFLD patients was 1.77 (95% CI 1.15, 2.39) lower than that in normal controls. We also found a significantly higher occurrence risk of NAFLD (OR = 1.33, 95% CI 1.20 to 1.48), NASH (OR = 2.42, 95% CI 1.27 to 3.57) and NAFLD-related significant fibrosis (OR = 1.56, 95% CI 1.34, 1.78) in sarcopenia subjects.

**Conclusions:**

SMI level in patients with NAFLD was lower than healthy people, and patients with sarcopenia have higher occurrence risk of NAFLD, as well as its advanced stages including NASH or NAFLD-related significant fibrosis. Further well-designed prospective studies are required to strengthen the arguments.

**Electronic supplementary material:**

The online version of this article (10.1007/s12072-019-09964-1) contains supplementary material, which is available to authorized users.

## Introduction

Nonalcoholic fatty liver disease (NAFLD) has gradually become one of the most common chronic liver diseases in the world, and approximately 25% of the adult population were affected [1]. It is expected that by the year 2025, NAFLD (Nonalcoholic fatty liver disease) will become the dominant cause of end-stage liver disease and liver transplantation [2, 3]. NAFLD contains many disease states, ranging from nonalcoholic fatty liver (NAFL) to nonalcoholic steatohepatitis (NASH). NASH (nonalcoholic steatohepatitis) may develop to liver fibrosis and hepatocellular carcinoma and bring a high occurrence risk of non-liver-associated complications such as cardiovascular disease [4]. Therefore, early identification and intervention of NAFLD patients who are at high risk for progressing to NASH and especially to NAFLD-related liver fibrosis may help reduce the burden associated with these diseases.

Skeletal muscle has been considered to be an important endocrine organ that secretes myokines and participates in postprandial glucose utilization, which is a crosstalk between muscle, adipose tissue, liver and other organs. Relative skeletal muscle mass, represented by skeletal muscle mass index (SMI), is the skeletal muscle mass (SMM) divided by height squared or weight. Several cohort and cross-sectional studies have indicated that SMI (skeletal muscle mass index) is associated with the Incidence rate of NAFLD [5–8]. In addition, some researchers found that a reduction in SMM might cause metabolic disorders and deteriorate NAFLD [9, 10]. Sarcopenia is a condition characterised by a general and progressive loss of strength and SMM, often associated with functional impairment, physical disability, and increased mortality [11]. In the past few years, many high-quality studies have emerged to explore the relationship between sarcopenia and the presence and severity of NAFLD [12, 13].

Recently, more and more studies are focusing on the relationship between relative skeletal muscle mass, sarcopenia and NAFLD, but the answer remains controversial. Some studies have reported that patients with sarcopenia have a lower risk of developing NAFLD [12, 14], while others have reached the opposite conclusion [10, 15]. In this meta-analysis, we aimed to quantify the association between SMI, sarcopenia and the presence and severity of NAFLD.

## Methods and materials

### Search strategy and literature selection

This systematic review and meta-analysis were conducted based on the Preferred Reporting Items for Systematic Reviews and Meta-Analyses (PRISMA) Statement (Table S4). We retrieved English relevant studies from PubMed, Embase database, the Web of Science and the Cochrane Library. The latest literature screened was published on December 20th, 2018. The electronic retrieval strategy included the following terms: (“NAFLD” OR “Nonalcoholic Fatty Liver Disease” OR “Nonalcoholic Steatohepatitis” OR “NASH”) AND (“Skeletal Muscle” OR “Skeletal Muscles”). Specific search strategies for each database can be found in supplementary materials. Furthermore, the reference lists of key articles were manually and independently reviewed.

### Literature inclusion and exclusion criteria

Studies inclusion criteria were as follows: (1) observational studies or cross-sectional studies; (2) providing a concrete analysis on the odds ratio (OR) that measuring the association between NAFLD、NASH、 NAFLD-related fibrosis and sarcopenia or the mean SMI in NAFLD cases and controls with 95% CI. Studies exclusion criteria were as follows: (1) cell or animal studies, reviews, comments and letters; (2) duplicated studies; (3) research on irrelevant topics; (4) without necessary data or information. The titles and abstracts of the studies were reviewed by two researchers (CC and XS) independently, and which met the inclusion criteria underwent full-text assessment. Discrepancies were resolved by the third reviewer. The information extracted from each selected study was as follows: first author, year of publication, country, study design, measurement of SMM, calculation of SMI, diagnosis of sarcopenia, diagnosis of NAFLD and fibrosis, population characteristics (disease stages and number of cases and controls), adjustment for confounding variables, adjusted OR with 95% CI and SMI with 95% CI.

### Article quality and bias assessment

The methodological quality of the enrolled cohort studies was evaluated by the Newcastle–Ottawa Scale (NOS) checklist and determined according to the selection of study groups, comparability of groups and ascertainment of the outcome. The methodological quality of the cross-sectional studies was evaluated by an 11-item checklist, which was known as Agency for Healthcare Research and Quality (AHRQ) (Table S2). If it was defined ‘YES’, the item would be scored ‘one point’; and the score would be a zero if it was defined ‘NO’ or ‘UNCLEAR’. The quality score of the study was graded as follows: low quality defined as 0–3; moderate quality defined as 4–7; high quality defined as 8–11. The supplementary material provides the bias assessment for the studies included.

### Statistical analysis

The outcome measure of the meta-analysis was conducted in five aspects. First, we calculated the weighted mean difference (WMD) of SMI between NAFLD patients and normal controls (NC). Subgroup analysis according to gender was performed. SMM measurement methods were divided into two types: bioimpedance analysis (BIA) and dual-energy X-ray absorptiometry (DEXA). We also performed subgroup analysis based on the different SMM measurement methods. Second, we evaluated the strength of association between the risk of NAFLD, NASH and sarcopenia. Further stratified analyses based on race and sample size were performed. Next, we assessed the risk of significant liver fibrosis in NAFLD patients with sarcopenia compared to NAFLD patients without sarcopenia. And subgroup analyses that based on race and whether the diagnosis of NAFLD-related fibrosis was invasive or non-invasive were performed. Relative skeletal muscle mass represented by SMI is the SMM divided by weight × 100%. We analyzed SMI as a continuous variable and calculated the pooled estimates of mean difference between NAFLD patients and NC. To analyze the strength of the association between NAFLD and sarcopenia, we calculated pooled adjusted OR (odds ratio) with 95% CIs.

The publication bias was evaluated by constructing a funnel plot of each study’s effect size against the standard error. Funnel plot asymmetry was evaluated by Egger’s test, and *p* value < 0.1 was defined as having significant publication bias. And we conducted Trim-and-fill analysis to evaluate the effect of publication bias on the interpretation of the results. Cochran Q test and *I*^2^ test were used to assess the heterogeneity between the studies. The low, medium or high heterogeneity was represented by 25%, 50% or 75% of the *I*^2^ value, respectively. The random effects model was selected if the p value was less than 0.1 or the *I*^2^ value was more than 50%; otherwise, the fixed effects model was chosen. All statistical analyses were performed by Stata (version 12.0).

## Results

### Study identification and literature characteristics

**Fig. 1:**
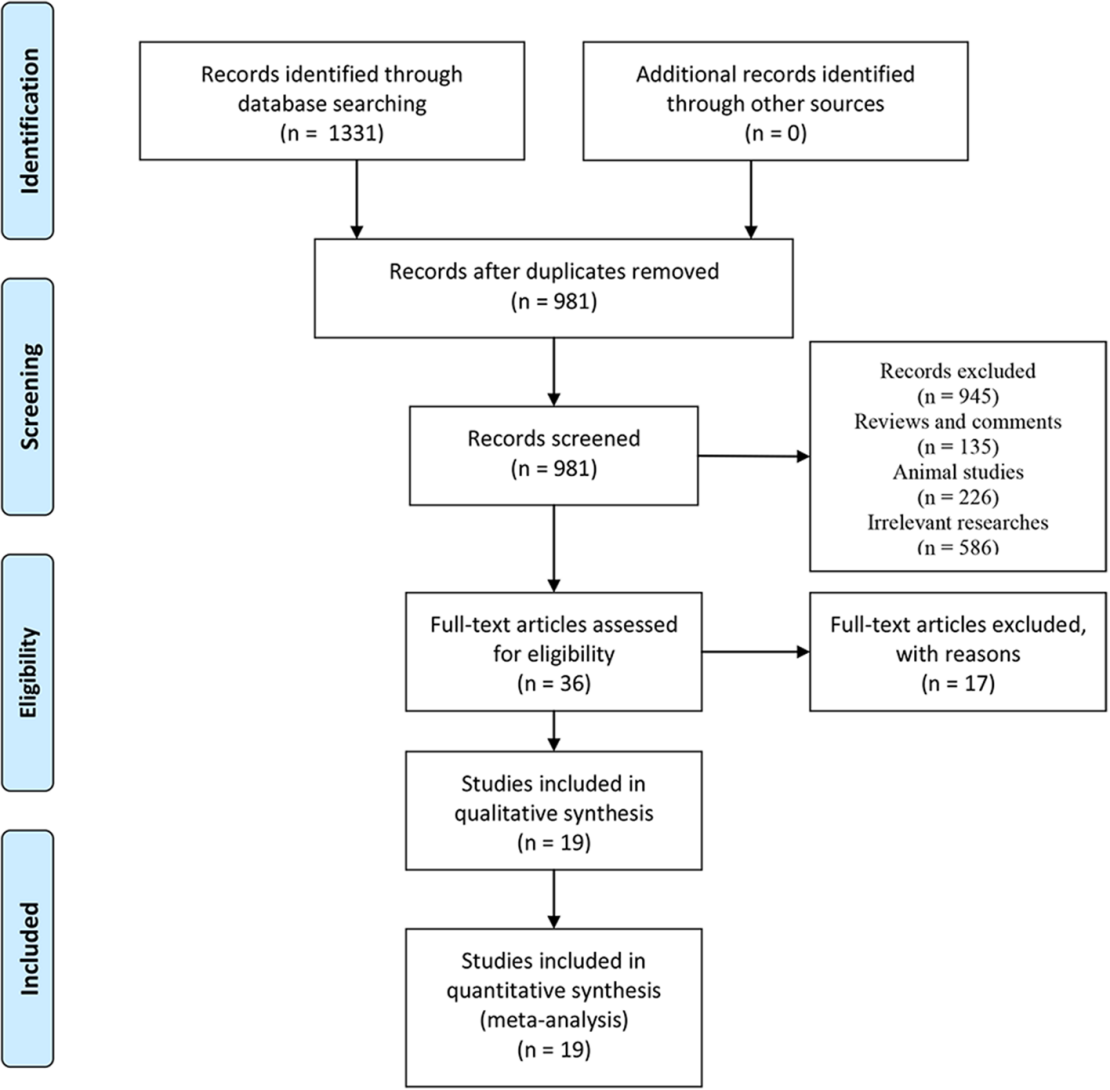
Flowchart showing the selection of articles included in the meta-analysis

A total of 1331 articles were retrieved, of which, 214 were from Pubmed, 540 were from Embase, 556 were from Web of Science and 21 were from Cochrane. After the exclusion of 350 duplicates, 135 reviews and comments, 226 animal studies, 586 irrelevant studies, and 17 articles without concrete data (Fig. 1), 19 articles were eventually included in our meta-analysis [5–10, 12–24]. Characteristics of all the studies included are listed in Table 1, among which, 2 were in retrospective cohort design and 17 were in a cross-sectional design. The bias risk of the cohort studies was assessed by NOS in Table S1, while that of the cross-sectional studies was assessed by AHRQ in Table S3.

**Table 1:** Characteristics of the included studies

Study	Design	Calculation of SMI; Diagnosis of sarcopenia	Diagnosis of NAFLD	Diagnosis of fibrosis	OR for NAFLD	Confounder adjustment	OR for advanced fibrosis	Confounder adjustment	SMI of Case N; Mean (SD)	SMI of Control; N; Mean (SD)
Lee [8], Korea	Cohort	SMI = SMM/BW; NA; estimated by BIA	By liver ultrasound	NA	NA	NA	NA	NA	NAFLD 597; 32.76 (1.86)	NC 3807; 34.23 (1.91)
Shen [12], America	Cross-sectional	SMI = SMM/Ht; (Man: ≤ 10.75 kg/m2; Woman: ≤ 6.75 kg/m2); estimated by BIA	By liver ultrasound	NA	1 (0.79–1.27)	NA	NA	NA	NA	NA
Kim [7], Korea	Cross-sectional	SMI = SMM/BW; NA; measured by DEXA	By FLI	NA	Assessed in man 1.35 (1.17–1.54); Assessed in woman 1.36 (1.18–1.55)	1, 6, 12, 13, 14, 15, 17, 23, 30	NA	NA	NAFLD 387; 26.77 (2.79)	NC 3348; 27.32 (4.43)
Peng [9], China	Cross-sectional	SMI = SMM/BW; (MAN: < 37.0%; woman: < 28%); SMI = SMM/Ht; (Man: < 10.76 kg/m2;woman: < 6.75 kg/m2) estimated by BIA	By liver ultrasound	NA	Mild NAFLD 1.41 (1.11–1.86); Moderate NAFLD 1.88 (1.5–2.37); Severe NAFLD 1.52 (1.14–2.04)	1, 3, 8, 14, 15, 22, 23, 27, 29, 35	NA	NA	NAFLD 1080;30.86 (3.86); Advanced NAFLD 740; 30.48 (3.63)	NC 1469; 32.11 (4.04); Mild NAFLD 342; 31.49 (3.97)
Kim [6], Korea	Cohort	SMI = SMM/BW; NA; estimated by BIA	By HSI	NA	NA	NA	NA	NA	NAFLD 2943;29.80 (2.53)	NC 12624; 31.10 (2.57)
Hong [13], Korea	Cross-sectional	SMI = SMM/BW; (Man: < 39.8%; woman: < 34.1%); measured by DEXA	LAI < 5HU	NA	5.16 (1.63–16.3)	1, 3, 6, 14, 15, 22, 23	NA	NA	NA	NA
Lee [14], Korea	Cross-sectional	SMI = SMM/BW; (Man: < 32.2%; woman: < 25.5%); measured by DEXA	By HSI, CNS, and LFS	NA	Assessed by HSI 1.18 (1.03 − 1.34); Assessed by CNS 1.19 (1.02 − 1.39); Assessed by LFS 1.22 (1.09 − 1.36)	1, 3, 6, 13, 14, 15	NA	NA	NA	NA
Koo [10], Korea	Cross-sectional	SMI = SMM/BW;(Man: < 29.0%; woman: < 22.9%) SMI = SMM/BMI;(Man: < 0.789; woman: < 0.512); estimated by BIA	By liver biopsy	By liver biopsy	Sarcopenia_Weight 1.53(0.50–4.65); Sarcopenia_BMI 1.27 (0.41–3.95)	1, 3	Sarcopenia_Weight 2.05 (1.01–4.16); Sarcopenia_BMI 2.24 (1.06–4.73)	1, 3, 4, 6, 9, 12, 13, 15, 24, 25	Advanced NAFLD 123; 26.59 (3.76)	Mild NAFLD 117; 28.29 (3.91)
Petta [15], Italy	Cross-sectional	SMI = SMM/BW; (Man: ≤ 37%; woman: ≤ 28); estimated by BIA	By liver biopsy	By liver biopsy	NA	NA	2.36 (1.15–4.84)	NA	NA	NA
Lee [16], Korea	Cross-sectional	SI = SMM/BMI; (Man: < 0.789; woman: < 0.521); measured by DEXA	By NFS, CNS, HSI	By NFS; By FIB-4	NA	NA	By NFS 1.49 (1.10–2.02) By FIB-4 1.37 (1.01–1.86)	1–21	NA	NA
Choe [5], Korea	Cross-sectional	SMI = SMA/BMI; (Man: < 8.37 cm2/(kg/m2); Woman: < 7.47 cm2/(kg/m2)); measured by CT	By liver ultrasound	NA	1.51 (1.15–1.99)	1, 3, 5, 7, 8, 13, 15, 26	NA	NA	NA	NA
Hashimoto [17], Japan	Cross-sectional	SMI = SMM/BW; NA; estimated by BIA	By transient elastography	NA	Assessed in man 1.25 (1.03–1.52)	1, 4, 15, 26, 27, 28	NA	NA	NAFLD 95; 38.42 (4.25)	NC 46; 43.10 (3.99)
Zhai [18], China	Cross-sectional	SMI = SMM/Ht; (Man: < 7.0 kg/m2; Woman: < 5.4 kg/m2); measured by DEXA	By liver ultrasound	NA	0.48 (0.31–0.74)	NA	NA	NA	NA	NA
Moon [19], Korea	Cross-sectional	SMI = SMM/BW; NA; estimated by BIA	By FLI ≥ 60	NA	NA	NA	NA	NA	NAFLD 1848; 38.2 (4.4)	NC 7717; 41.30 (4.00)
Choi [20]. Korea	Cross-sectional	SMI = SMM/BW; NA/NA	By liver ultrasound	NA	Assessed in woman 2.25 (1.66–3.04)	1, 5, 8, 9, 27, 32	NA	NA	NA	NA
Kang [21], Korea	Cross-sectional	SMI = SMM/BW; NA; estimated by BIA	By liver ultrasound	By FIB-4	NA	NA	1.5977 (1.27–2.01)	3; 30	NA	NA
Kim [22], Korea	Cross-sectional	SMI = SMM/BW; patients with lowest quartile; estimated by BIA	By liver biopsy	NA	4.258 (1.273–14.246)	1, 6, 30, 33	NA	NA	NA	NA
Kwanten [23], Belgium	Cross-sectional	SMI = SMM/BW; NA; estimated by BIA or TPA or CT	By liver biopsy	By liver biopsy	NA	NA	1.66 (0.70–3.94)	NA	NA	NA
Wijarnpreecha [24], America	Cross-sectional	SMI; NA; estimated by BIA	By liver ultrasound	By NFS	1.24 (1.03–1.48)	1, 3, 29, 30, 31	1.79 (1.18–2.72)	30, 31	NA	NA

1: age; 2: age × SI; 3: gender; 4: BMI; 5: waist circumference; 6: HOMA-IR; 7: fasting glucose; 8: total cholesterol; 9: triglyceride; 10: aspartate aminotransferase; 11: alanine aminotransferase; 12: diabetes status; 13: hypertension; 14: exercise; 15: smoking; 16: estimated glomerular filtration rate; 17: drinking; 18: residence; 19: history of cerebrovascular and coronary heart disease; 20: chronic obstructive pulmonary disease; 21: malignancy; 22: high sensitivity C-reactive protein; 23: 25-hydroxyvitamin D parameter levels; 24: platelet; 25: albumin levels; 26: triglycerides/HDL-C ratio; 27: hemoglobin A1c; 28: gamma-glutamyl transferase; 29: ethnicity; 30: metabolic risk factors; 31: vitamin D deficiency; 32: blood pressure; 33: fat mass; 34: white blood cell; 35: serum uric acid

*NAFLD* nonalcoholic fatty liver disease, *NC* normal control, *N* number, *SD* standard deviation, *SMI* skeletal muscle index, *SI* sarcopenia index, *SMA* skeletal muscle area, *SMM* skeletal muscle mass, *BW* body weight, *Ht* height, *BMI* body mass index, *OR* odds ratio, *FLI* fatty liver index, *HSI* hepatic steatosis index, *LAI* liver attenuation index, *CNS* comprehensive NAFLD score, *LFS* liver fat score, *NFS* NAFLD fibrosis score, *FIB-4* fibrosis index based on the 4 factor, *CAP* controlled attenuation, *HOMA-IR* homeostasis model assessment of insulin resistance, *HDL* high density lipoprotein

### Decreased SMI in NAFLD

**Fig. 2:**
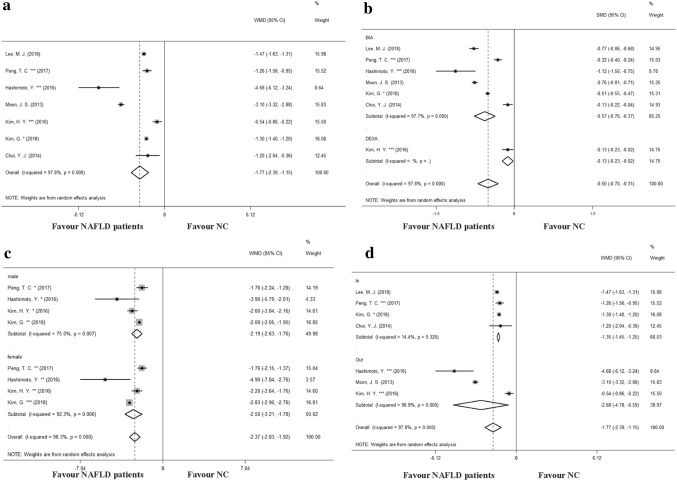
Meta-analysis of skeletal muscle index (SMI) in nonalcoholic fatty liver disease (NAFLD). **a** A pooled weighted mean difference (WMD) of SMI in overall seven individual studies between NAFLD patients and normal people. **b** A pooled WMD of SMI in subgroup analysis based on the different SMM measurement methods. **c** A pooled WMD of SMI in subgroup analysis according to gender. **d** Sensitivity analysis according to whether the study was in or out of the funnel plot. For each estimate, the grey shaded area is the weight of the estimate in proportion to the overall effect. *SMI* skeletal muscle index, *NAFLD* nonalcoholic fatty liver disease, *WMD* weighted mean difference, *NC* normal control, *SMM* skeletal muscle mass, *DEXA* dual energy X-ray absorptiometry, *BIA* bioimpedance analysis

In a pooled analysis of seven individual studies, the group of NAFLD patients (*N* = 7934) showed a lower mean SMI than the group of NC (*N* = 29,533), with WMD − 1.77 (95% CI − 2.39, − 1.15) and statistically significant between-study heterogeneity (*I*^2^ = 97.8%, *p* = 0.000) (Fig. 2a). In this part of the analysis, no significant publication bias was found (*p* = 0.835) (Figure S1). We performed a subgroup analysis based on the different SMM measurement methods (Fig. 2b). Results showed that in the bioimpedance analysis (BIA) diagnostic subgroup, the SMI of patients with NAFLD was 0.57 (95% CI 0.37, 076) lower than that of the control group, while in the dual energy X-ray absorptiometry (DEXA) subgroup, the SMI of patients with NAFLD was 0.13 (95% CI 0.02, 023) lower than the control group. Besides, subgroup analysis according to gender showed that the WMD (weighted mean difference) in male subgroup and female subgroup was − 2.19 (95% CI − 2.63, − 1.76) and − 2.50 (95% CI − 3.21, − 1.92), respectively (Fig. 2b). Further sensitivity analysis indicated that the SMI level in patients with NAFLD was lower than that of the NC group, while the WMD between the two groups was − 1.35 (95% CI − 1.45, − 1.25), with no significant between-study heterogeneity (*I*^2^ = 14.4%, *p* = 0.320) (Fig. 2c).

### Increased risk of NAFLD in sarcopenia

**Fig. 3:**
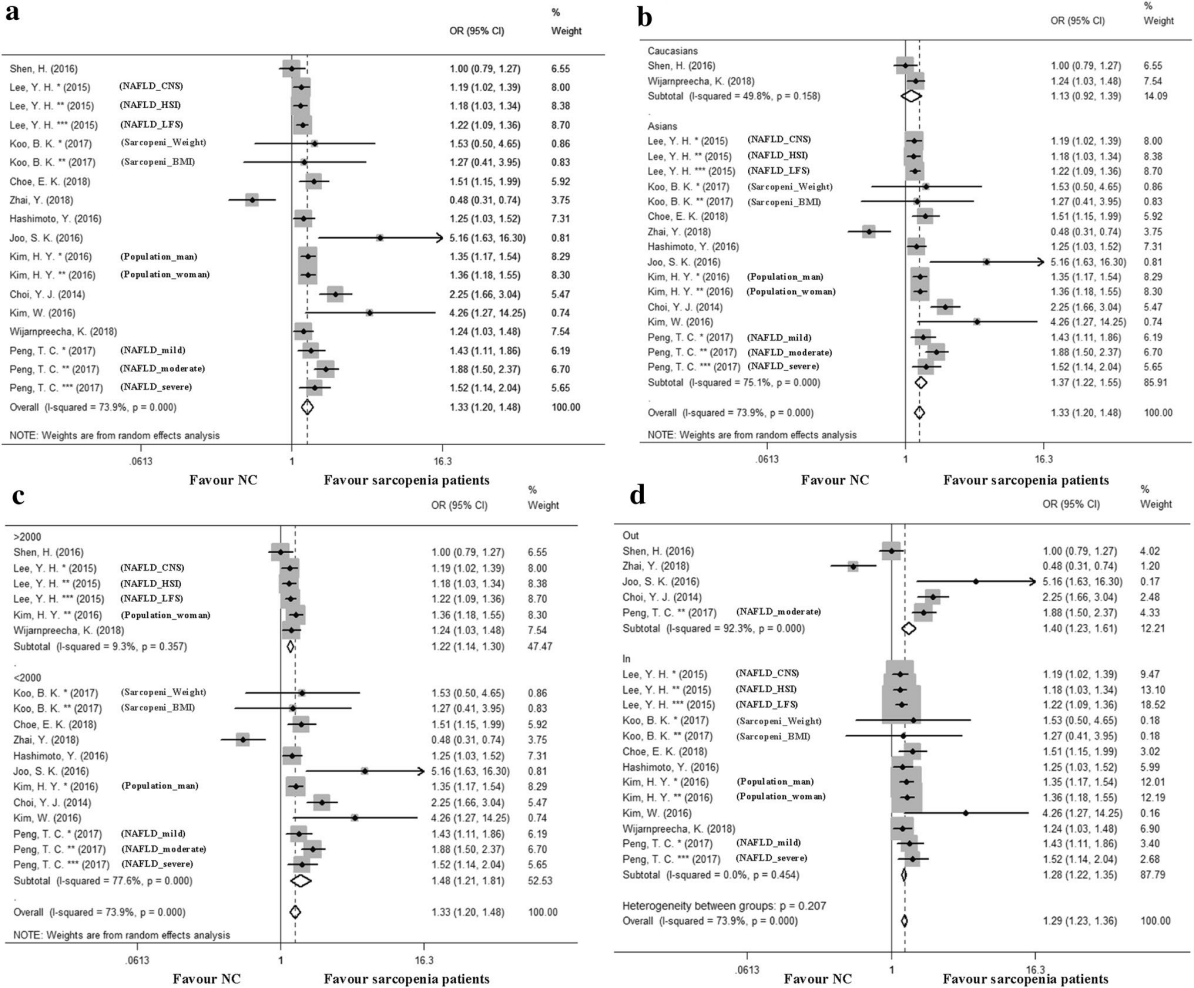
Meta-analysis of sarcopenia in nonalcoholic fatty liver disease (NAFLD). **a** A pooled odds ratio (OR) of NAFLD prevalence between sarcopenia patients and normal control (NC). **b** Subgroup analysis according to race. **c** Subgroup analysis based on the sample size. **d** Sensitivity analysis according to whether the study was in or out of the funnel plot. For each estimate, the grey shaded area is the weight of the estimate in proportion to the overall effect. *NAFLD* nonalcoholic fatty liver disease, *NC* normal control, *OR* odds ratio, *HSI* hepatic steatosis index, *CNS* comprehensive NAFLD score, *NFS* NAFLD fibrosis score, *BMI* body mass index

18 studies (*N* = 48,079 participants) assessed the adjusted OR of NAFLD prevalence between sarcopenia patients and NC (Fig. 3a). Overall, the pooled adjusted OR suggested that sarcopenia was strongly associated with an increased occurrence risk of NAFLD (OR = 1.33, 95% CI 1.20, 1.48), with statistically significant between-study heterogeneity (*I*^2^ = 73.9%, *p* = 0.000). There was no evident publication bias (*p* = 0.113) (Figure S2). We then performed stratified analyses among Caucasians and Asians. An increased risk of NAFLD was observed to be related to sarcopenia in the Asian population (OR = 1.37, 95% CI 1.22, 1.55), with significant between-study heterogeneity (*I*^2^ = 73.9%, *p* = 0.000) (Fig. 3b). Moreover, we conducted stratified analyses based on the sample size. In the subgroup among which each study enrolled more than 2000 samples, an increased risk of NAFLD was observed in sarcopenia patients (OR = 1.22, 95% CI 1.14, 1.30), with no significant between-study heterogeneity (*I*^2^ = 9.3%, *p* = 0.357) (Fig. 3c). Further in the sensitivity analysis pooling the studies within the scope of the funnel, OR was 1.28 (95% CI 1.26, 1.23), with no significant between-study heterogeneity (*I*^2^ = 0.0%, *p* = 0.454) (Fig. 3d).

**Fig. 4:**
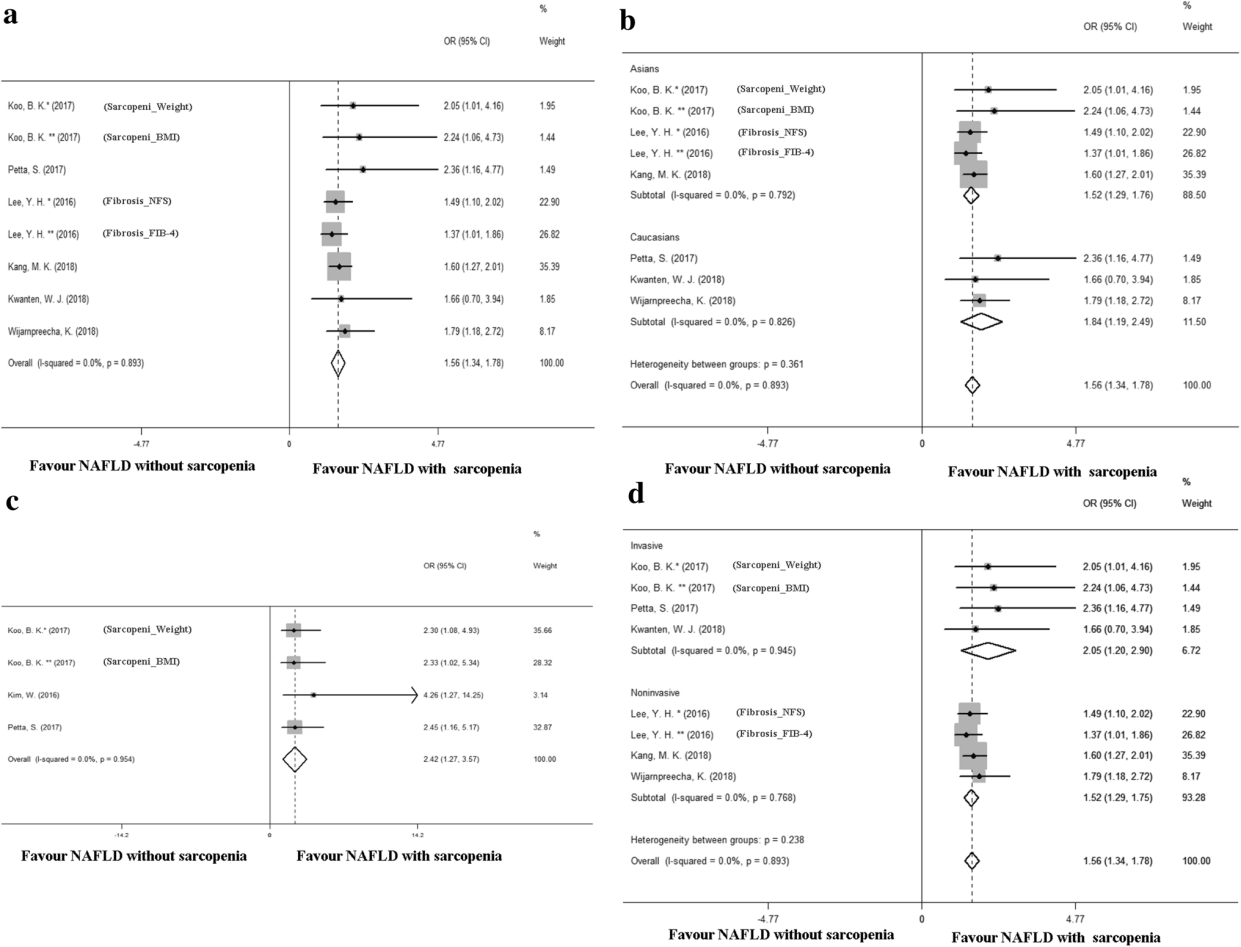
Meta-analysis of sarcopenia in nonalcoholic fatty liver disease (NAFLD). **a** A pooled odds ratio (OR) of NAFLD-related significant fibrosis between sarcopenia patients and normal control (NC). **b** Subgroup analysis according to race. **c** In NAFLD patients, a pooled OR of NASH between sarcopenia patients and control. **d** In analyzing the association in patients with sarcopenia and NAFLD-related significant fibrosis. Subgroup analysis was performed based on the diagnostic methods. *NAFLD* nonalcoholic fatty liver disease, *OR*: odds ratio, *NFS* NAFLD fibrosis score, *FIB-4* fibrosis index based on the 4 factor, *BMI* body mass index

Four studies (*N* = 469 participants) indicated a higher risk of NASH prevalence in sarcopenia patients (OR = 2.42, 95% CI 1.27 to 3.57) (Fig. 4c), with no between-study heterogeneity (*I*^2^ = 0.0%, *p* = 0.954). Significant publication bias (*p* = 0.049) was found in this analysis (Figure S3A). However, further analysis with trim-and-fill test showed that this publication bias did not impact the estimates (Figure S3B).

### Increased risk of NAFLD-related significant fibrosis in sarcopenia

A pooled analysis of 8 studies (*N* = 25,434 patients) showed a higher prevalence of NAFLD-related significant fibrosis in sarcopenia patients compared to NAFLD patients without sarcopenia, with pooled adjusted OR 1.56 (95% CI 1.34, 1.78) (Fig. 4a). In this analysis, no between-study heterogeneity was observed (*I*^2^ = 0.0%, *p* = 0.893), while publication bias did exist according to Egger’s test (*p* = 0.047) (Figure S4A). However, the further trim-and-fill analysis showed that the pooled log OR before and after trimming was 0.472 (95% CI 0.372, 0.620) and 0.433 (95% CI 0.333, 0.561), showing that the publication bias had little impact on the interpretation of the results (Figure S4B). We then performed stratified analyses among Caucasians and Asians. An increased risk of NAFLD-related significant fibrosis was observed in sarcopenia patients in both the Asian population (OR = 1.52, 95% CI 1.29, 1.76, *I*^2^ = 0.0%) and the Caucasian population (OR = 1.84, 95% CI 1.19, 2.49, *I*^2^ = 0.0%) (Fig. 4b). Subsequently, we conducted a subgroup analysis based on whether the diagnosis of NAFLD reached invasively or non-invasively. We found that in the invasively diagnosed subgroup, sarcopenia patients had a higher risk of NAFLD-related significant fibrosis (OR = 2.05, 95% CI 1.20, 2.90, *I*^2^ = 0.0%) than in the non-invasively diagnosed subgroup (OR = 1.52, 95% CI 1.29, 1.75, *I*^2^ = 0.0%) (Fig. 4d).

## Discussion

Our study performed a comprehensive evaluation of the epidemiologic data focusing on the association between SMI, sarcopenia and the presence and severity of NAFLD. We conducted four separate meta-analyses to better quantify the relations. This meta-analysis is hitherto the first study to explore whether the SMI of NAFLD patients differed from that of normal people. Besides, we first offered a pooled estimate and quantitative assessment of the clinical risk of NAFL, NASH and NAFLD-related significant fibrosis in patients with sarcopenia. We demonstrated that, when compared with normal people, NAFLD patients had a lower mean SMI, with WMD − 1.77 (95% CI − 2.39 to − 1.15). The difference turned out to be more obvious in female subgroup than in male subgroup, and the difference in the BIA subgroup was greater than in the DEXA subgroup. However, the data of male and female research, BIA and DEXA research is consistent in terms of trends and meanings, indicating that gender and SMM measurement method does not affect the conclusions of the overall pooled results. As was reported before [18], after adjusting for some confounders in men, for every 1% increase in SMI, the odds ratio for fatty liver disease was 0.80 (95% CI 0.64–0.97, *p* = 0.021). These important findings suggested that the lower the SMI one has, the higher the risk of developing NAFLD. Consequently, we showed that sarcopenia subjects having a significantly increased risk of NAFLD (OR = 1.33, 95% CI 1.20 to 1.48). The association was stronger in Asian participants than in Caucasian participants, but the meaning of the results is consistent, which indicated that the race does not affect the overall pooled results. In the robust subgroup with a sample size greater than 2000, the pooled OR was 1.22 (95% CI 1.14, 1.30) with no significant between-study heterogeneity (*I*^2^ = 9.3%, *p* = 0.357), indicating that when the sample size of the study is larger, the results tend to be more consistent and stable [7, 12, 15, 24]. A higher risk of NASH was seen in NAFLD patients with sarcopenia patients than in NAFLD patients without sarcopenia, with an estimated 2.42-fold. Besides, the risk of NAFLD-related significant fibrosis was higher in NAFLD patients with sarcopenia than in the NAFLD patients without sarcopenia (OR = 1.56, 95% CI 1.34, 1.78). The pooled results were more remarkable in Caucasian participants than in Asian participants and in invasively diagnosed subgroup than in noninvasively diagnosed subgroup. However, the results of the Caucasion subgroup and the Asian subgroup had the same trend and significance, and the results of the invasive subgroup and the non-invasive subgroup have the same meaning, indicating that the race and diagnostic methods do not affect the interpretation of overall pooled data. The results indicated that sarcopenia was associated with a higher risk of developing NAFLD, especially of its advanced stages such as NASH or liver fibrosis. Two retrospective cohort studies included in our analysis reported that decreased muscle mass was an independent predictor of NAFLD in both male and female [8] and an increasing SMI over time was beneficial for preventing both the occurrence and the progress of NAFLD [6]. It is worth noting that some studies have suggested that patients with NASH or fibrosis have a relatively poor prognosis [25]. The status of skeletal muscle mass in patients with NAFLD deserves attention and timely interventions such as nutrition supplement and exercise should be taken to guard against further development to NASH, fibrosis and other events of poor prognosis. In fact, the mechanisms by which low SMI and sarcopenia increase the occurrence risk of NAFLD, NASH and even significant fibrosis are not completely elucidated. Possible explanations currently under discussion are summarized as the following.

First, skeletal muscle plays a crucial role in insulin signaling as a primary tissue responsible for insulin-mediated glucose disposal [26]. A reduction in skeletal muscle mass may give rise to insulin resistance and dysglycemia, ultimately leading to NAFLD and its significant patterns [27]. Second, oxidative stress and chronic inflammation have been reported to cause muscle atrophy and lead to stress responses in hepatocytes, leading to the progression of NASH- and NAFLD–related liver fibrosis [28, 29]. Third, skeletal muscle is an endocrine organ secreting peptides called myokines, such as interleukin-6, which has a protective effect from developing NAFLD [30]. Irisin, another myokines, plays a critical role in fatty acid β-oxidation in the liver [31]. Hence, it is plausible that decreased skeletal muscle could be the cause of NAFLD incidence due to reduced secretion of various salutary myokines. Fourth, growing evidence has revealed a close association between low SMM and decline of vitamin D [32].

Several limitations need to be considered when interpreting the results of our work. First, despite our efforts to analyze comprehensively and accurately, some related studies might be omitted. Second, considering that most of the 19 articles we analyzed were in cross-sectional design and only 2 of them were retrospective cohort studies, we could not fully determine the cause-effect relationship between sarcopenia and NAFLD. Third, the definition of SMI varies from study to study. Most studies defined SMI as SMM divided by body weight [6–10, 13, 15, 16, 18–23], while others adopted SMM divided by BMI or height [5, 9, 10, 12, 14, 17, 33]. Fourth, although liver biopsy is still the gold standard for ascertaining NAFL and NASH, some of our original studies used imaging methods and predictive models, which are less sensitive in the detection of mild steatosis. And although the definition of significant fibrosis in classification methods and surrogate indices is different, these methods are also well validated.

In conclusion, our meta-analysis indicated that SMI level in patients with NAFLD was lower than healthy people and sarcopenia is associated with NAFLD and particularly the most advanced forms of NASH and fibrosing NAFLD. Restoration of relative skeletal muscle mass may help prevent the onset of NAFLD or its progression, both in normal people and patients with sarcopenia. Clinicians need to bear in mind a potential diagnosis of NAFLD when confronting patients with low relative skeletal muscle mass or sarcopenia. When the diagnosis is made, lifestyle intervention, as the cornerstone management, can be implemented as soon as possible. Since most of the included studies are cross-sectional, the directionality of our results cannot be completely ascertained. Further well-designed prospective studies are required to consolidate these conclusions.

## Electronic supplementary material

Below is the link to the electronic supplementary material.

Supplementary material 1 (DOC 573 kb)

## Abbreviations

NAFLD: Nonalcoholic fatty liver disease
PRISMA: Preferred reporting items for systematic reviews and meta-analyses
NASH: Nonalcoholic steatohepatitis
NC: Normal control
SD: Standard deviation
WMD: Weighted mean difference
SMI: Skeletal muscle index
SI: Sarcopenia index
SMA: Skeletal muscle area
SMM: Skeletal muscle mass
AHRQ: Agency for healthcare research and quality
BW: Body weight
BMI: Body mass index
OR: Odds ratio
