# Supplementary material for: Relationship between relative skeletal muscle mass and nonalcoholic fatty liver disease: a systematic review and meta-analysis
Source: Hepatol Int. 2019 Jul 9;14(1):115–26. doi: 10.1007/s12072-019-09964-1 (PMC6994447; doi:10.1007/s12072-019-09964-1)
Supplement: Supplementary file 1 — Supplementary material 1 (DOC 573 kb) [file 12072_2019_9964_MOESM1_ESM.doc]

**Search strategy:**

**Pubmed:** ((((Non alcoholic Fatty Liver Disease[Title/Abstract] OR NAFLD[Title/Abstract] OR Nonalcoholic Fatty Liver Disease[Title/Abstract] OR Fatty Liver, Nonalcoholic[Title/Abstract] OR Fatty Livers, Nonalcoholic[Title/Abstract] OR Liver, Nonalcoholic Fatty[Title/Abstract] OR Livers, Nonalcoholic Fatty[Title/Abstract] OR Nonalcoholic Fatty Liver；Nonalcoholic Fatty Livers[Title/Abstract] OR Nonalcoholic Steatohepatitis[Title/Abstract] OR Nonalcoholic Steatohepatitides[Title/Abstract] OR Steatohepatitides, Nonalcoholic[Title/Abstract] OR Steatohepatitis, Nonalcoholic[Title/Abstract] OR Non-alcoholic steatohapatitis[Title/Abstract] OR non alcoholic steatohapatitis[Title/Abstract] OR NASH[Title/Abstract] OR Non-alcoholic Fatty Liver Disease[Title/Abstract])) OR Non-alcoholic Fatty Liver Disease[MeSH Terms]）)) AND ((((Skeletal Muscle[Title/Abstract]) OR Muscles, Skeletal[Title/Abstract]) OR Skeletal Muscles[Title/Abstract]) OR "Muscle, Skeletal"[Mesh]))

**Embase:** ( 'nonalcoholic fatty liver'/exp OR 'non alcoholic fatty liver disease':ab,ti OR 'nafld':ab,ti OR 'nonalcoholic fatty liver disease':ab,ti OR 'fatty liver, nonalcoholic':ab,ti OR 'fatty livers, nonalcoholic':ab,ti OR 'liver, nonalcoholic fatty':ab,ti OR 'livers, nonalcoholic fatty':ab,ti OR 'nonalcoholic fatty liver':ab,ti OR 'nonalcoholic fatty livers':ab,ti OR 'nonalcoholic steatohepatitis':ab,ti OR 'nonalcoholic steatohepatitides':ab,ti OR 'steatohepatitides, nonalcoholic':ab,ti OR 'steatohepatitis, nonalcoholic':ab,ti OR 'non-alcoholic steatohapatitis':ab,ti OR 'non alcoholic steatohapatitis':ab,ti OR 'nash':ab,ti OR 'non-alcoholic fatty liver disease':ab,ti) and ('skeletal muscle':ab,ti OR 'muscles, skeletal':ab,ti OR 'skeletal muscles':ab,ti OR 'skeletal muscle'/exp)

**Web of Science:** (Non alcoholic Fatty Liver Disease OR NAFLD OR Nonalcoholic Fatty Liver Disease OR Fatty Liver, Nonalcoholic OR Fatty Livers, Nonalcoholic OR Liver, Nonalcoholic Fatty OR Livers, Nonalcoholic Fatty OR Nonalcoholic Fatty Liver OR Nonalcoholic Fatty Livers OR Nonalcoholic Steatohepatitis OR Nonalcoholic Steatohepatitides OR Steatohepatitides, Nonalcoholic OR Steatohepatitis, Nonalcoholic OR Non-alcoholic steatohapatitis OR non alcoholic steatohapatitis OR NASH OR Non-alcoholic Fatty Liver Disease) AND TOPIC: (Skeletal Muscle OR Muscles, Skeletal OR Skeletal Muscles OR Muscle, Skeletal)

**Cochrane:** (( MeSH descriptor: [Non-alcoholic Fatty Liver Disease] explode all trees) OR (Non alcoholic Fatty Liver Disease OR NAFLD OR Nonalcoholic Fatty Liver Disease OR Fatty Liver, Nonalcoholic OR Fatty Livers, Nonalcoholic OR Liver, Nonalcoholic Fatty OR Livers, Nonalcoholic Fatty OR Nonalcoholic Fatty Liver OR Nonalcoholic Fatty Livers OR Nonalcoholic Steatohepatitis OR Nonalcoholic Steatohepatitides OR Steatohepatitides, Nonalcoholic OR Steatohepatitis, Nonalcoholic OR Non-alcoholic steatohapatitis OR non alcoholic steatohapatitis OR NASH OR Non-alcoholic Fatty Liver Disease):ti,ab,kw) ) AND (( MeSH descriptor: [Muscle, Skeletal] explode all trees) OR ((skeletal muscle):ti,ab,kw OR (Muscles, Skeletal):ti,ab,kw OR (Skeletal Muscles):ti,ab,kw OR (Muscle, Skeletal):ti,ab,kw))

**Table S1** Methodological quality of included cohort studies assessed using a method based in the 9-star Newcastle-Ottawa Scale

| Year | First author | Selection | | | | Comparability | Outcome | Total |
| --- | --- | --- | --- | --- | --- | --- | --- | --- |
|  |  | Representativeness of the exposed cohort | Selection of the non-exposed cohort | Ascertainment of exposure | Demonstration that outcome of interest was not present at start of study | adjusted OR |  |  |
| 2018 | Lee (1) | ** | * | * | 0 | ** | *** | 9 |
| 2018 | Kim (2) | ** | * | * | 0 | ** | *** | 9 |

* represents one point, ** represents two points, *** represents three points. OR: odds ratio**.**

**Table S2 Agency for Healthcare Research and Quality (AHRQ)**

| Item | Yes | No | Unclear |
| --- | --- | --- | --- |
| 1) Define the source of information (survey, record review) |  |  |  |
| 2) List inclusion and exclusion criteria for exposed and unexposed subjects (cases and controls) or refer to previous publications |  |  |  |
| 3) Indicate time period used for identifying patients |  |  |  |
| 4) Indicate whether or not subjects were consecutive if not population-based |  |  |  |
| 5) Indicate if evaluators of subjective components of study were masked to other aspects of the status of the participants |  |  |  |
| 6) Describe any assessments undertaken for quality assurance purposes (e.g., test/retest of primary outcome measurements) |  |  |  |
| 7) Explain any patient exclusions from analysis |  |  |  |
| 8) Describe how confounding was assessed and/or controlled. |  |  |  |
| 9) If applicable, explain how missing data were handled in the analysis |  |  |  |
| 10) Summarize patient response rates and completeness of data collection |  |  |  |
| 11) Clarify what follow-up, if any, was expected and the percentage of patients for which incomplete data or follow-up was obtained |  |  |  |

**Table S3** Methodological quality of included cross-sectional studies assessed by 11-item checklist of Agency for Healthcare Research and Quality

|  | (3) | (4) | (5) | (6) | (7) | (8) | (9) | (10) | (11) | (12) | (13) | (14) | (15) | (16) | (17) | (18) | (19) |
| --- | --- | --- | --- | --- | --- | --- | --- | --- | --- | --- | --- | --- | --- | --- | --- | --- | --- |
| 1) | 1 | 1 | 1 | 1 | 1 | 1 | 1 | 1 | 1 | 1 | 1 | 1 | 1 | 1 | 0 | 1 | 1 |
| 2) | 1 | 1 | 1 | 1 | 1 | 1 | 1 | 1 | 0 | 0 | 0 | 0 | 1 | 0 | 0 | 1 | 0 |
| 3) | 1 | 1 | 1 | 1 | 1 | 0 | 0 | 1 | 1 | 1 | 1 | 1 | 1 | 0 | 0 | 0 | 0 |
| 4) | 1 | 1 | 1 | 1 | 1 | 1 | 1 | 1 | 1 | 0 | 0 | 1 | 0 | 1 | 0 | 0 | 1 |
| 5) | 0 | 0 | 0 | 0 | 0 | 0 | 0 | 0 | 0 | 0 | 0 | 0 | 0 | 0 | 0 | 0 | 0 |
| 6) | 1 | 1 | 1 | 1 | 1 | 1 | 1 | 1 | 1 | 1 | 1 | 1 | 1 | 1 | 1 | 1 | 1 |
| 7) | 1 | 1 | 1 | 1 | 1 | 1 | 1 | 1 | 1 | 1 | 1 | 1 | 0 | 0 | 0 | 0 | 0 |
| 8) | 1 | 1 | 1 | 1 | 1 | 1 | 1 | 1 | 1 | 1 | 1 | 1 | 1 | 1 | 1 | 1 | 1 |
| 9) | 0 | 1 | 1 | 1 | 1 | 1 | 1 | 1 | 1 | 0 | 0 | 1 | 0 | 0 | 0 | 0 | 0 |
| 10) | 0 | 1 | 1 | 1 | 1 | 1 | 1 | 1 | 1 | 1 | 1 | 1 | 0 | 0 | 0 | 0 | 0 |
| 11) | 0 | 0 | 0 | 0 | 0 | 0 | 0 | 0 | 0 | 0 | 0 | 0 | 0 | 0 | 0 | 0 | 0 |
| Sum | 7 | 9 | 9 | 9 | 9 | 8 | 8 | 9 | 8 | 6 | 6 | 8 | 5 | 4 | 2 | 4 | 4 |

The methodological quality of included cross-sectional studies was assessed using an 11-item checklist which was recommended by Agency for Healthcare Research and Quality (AHRQ). An item would be scored ‘0’ if it was answered ‘NO’ or ‘UNCLEAR’; if it was answered ‘YES’, then the item scored ‘1’. Article quality was assessed as follows: low quality = 0 - 3; moderate quality = 4 - 7; high quality = 8 - 11.

**Reference**

1. Lee MJ, Kim EH, Bae SJ, Kim GA, Park SW, Choe J, Jung CH, et al. Age-Related Decrease in Skeletal Muscle Mass Is an Independent Risk Factor for Incident Nonalcoholic Fatty Liver Disease: A 10-Year Retrospective Cohort Study. Gut Liver 2018.

2. Kim G, Lee SE, Lee YB, Jun JE, Ahn J, Bae JC, Jin SM, et al. Relationship Between Relative Skeletal Muscle Mass and Nonalcoholic Fatty Liver Disease: A 7-Year Longitudinal Study. Hepatology 2018;68:1755-1768.

3. Shen H, Liangpunsakul S. Association between sarcopenia and prevalence of nonalcoholic fatty liver disease: A cross-sectional study from the third national health and nutrition examination survey. Gastroenterology 2016;150:S1143-S1144.

4. Kim HY, Kim CW, Park CH, Choi JY, Han K, Merchant AT, Park YM. Low skeletal muscle mass is associated with non-alcoholic fatty liver disease in Korean adults: The Fifth Korea National Health and Nutrition Examination Survey. Hepatobiliary and Pancreatic Diseases International 2016;15:39-47.

5. Peng TC, Wu LW, Chen WL, Liaw FY, Chang YW, Kao TW. Nonalcoholic fatty liver disease and sarcopenia in a Western population (NHANES III): The importance of sarcopenia definition. Clinical Nutrition 2017.

6. Joo SK, Kim W, Oh S. Relationship between appendicular sarcopenia and nonalcoholic fatty liver disease in Korean population. Journal of Hepatology 2016;64:S496.

7. Lee YH, Jung KS, Kim SU, Yoon HJ, Yun YJ, Lee BW, Kang ES, et al. Sarcopaenia is associated with NAFLD independently of obesity and insulin resistance: Nationwide surveys (KNHANES 2008-2011). Journal of Hepatology 2015;63:486-493.

8. Koo BK, Kim D, Joo SK, Kim JH, Chang MS, Kim BG, Lee KL, et al. Sarcopenia is an independent risk factor for non-alcoholic steatohepatitis and significant fibrosis. Journal of Hepatology 2017;66:123-131.

9. Petta S, Ciminnisi S, Di Marco V, Cabibi D, Cammà C, Licata A, Marchesini G, et al. Sarcopenia is associated with severe liver fibrosis in patients with non-alcoholic fatty liver disease. Alimentary Pharmacology and Therapeutics 2017;45:510-518.

10. Lee YH, Kim SU, Song K, Park JY, Kim DY, Ahn SH, Lee BW, et al. Sarcopenia is associated with significant liver fibrosis independently of obesity and insulin resistance in nonalcoholic fatty liver disease: Nationwide surveys (KNHANES 2008-2011). Hepatology 2016;63:776-786.

11. Choe EK, Kang HY, Park B, Yang JI, Kim JS. The association between nonalcoholic fatty liver disease and CT-measured skeletal muscle mass. Journal of Clinical Medicine 2018;7.

12. Hashimoto Y, Osaka T, Fukuda T, Tanaka M, Yamazaki M, Fukui M. The relationship between hepatic steatosis and skeletal muscle mass index in men with type 2 diabetes. Endocrine Journal 2016;63:877-884.

13. Zhai Y, Xiao Q, Miao J. The Relationship between NAFLD and Sarcopenia in Elderly Patients. Canadian Journal of Gastroenterology and Hepatology 2018.

14. Moon JS, Yoon JS, Won KC, Lee HW. The role of skeletal muscle in development of nonalcoholic fatty liver disease. Diabetes and Metabolism Journal 2013;37:278-285.

15. Choi YJ, Kim SK, Kwak JJ, Park SW, Lee EJ, Huh KB. Age-related skeletal muscle loss as an independent predictor of NAFLD risk in Korean women with type 2 diabetes. Diabetes Research and Clinical Practice 2014;106:S162-S163.

16. Kang MK, Park JG, Kim MC, Park SY, Lee HJ, Tak WY, Kweon YO, et al. Sarcopenia is associated with advanced liver fibrosis in patients with non-alcoholic fatty liver disease. Hepatology 2018;68:1306A.

17. Kim W, Koo BK, Joo SK, Kim JH, Park SC. Sarcopenia is an independent risk factor for biopsyproven non-alcoholic steatohepatitis. Journal of Hepatology 2016;64:S502.

18. Kwanten WJ, De Fré C, De Fré M, Vonghia L, Vanwolleghem T, Michielsen PP, De Beeck BO, et al. Sarcopenia is less prevalent in an obese population with NAFLD compared to patients with obesity alone, but increases with severity of disease. Hepatology 2018;68:1282A-1283A.

19. Wijarnpreecha K, Scribani M, Kim D. Associations between sarcopenia and nonalcoholic fatty liver disease and advanced fibrosis in the United States. Journal of Hepatology 2018;68:S827-S828.


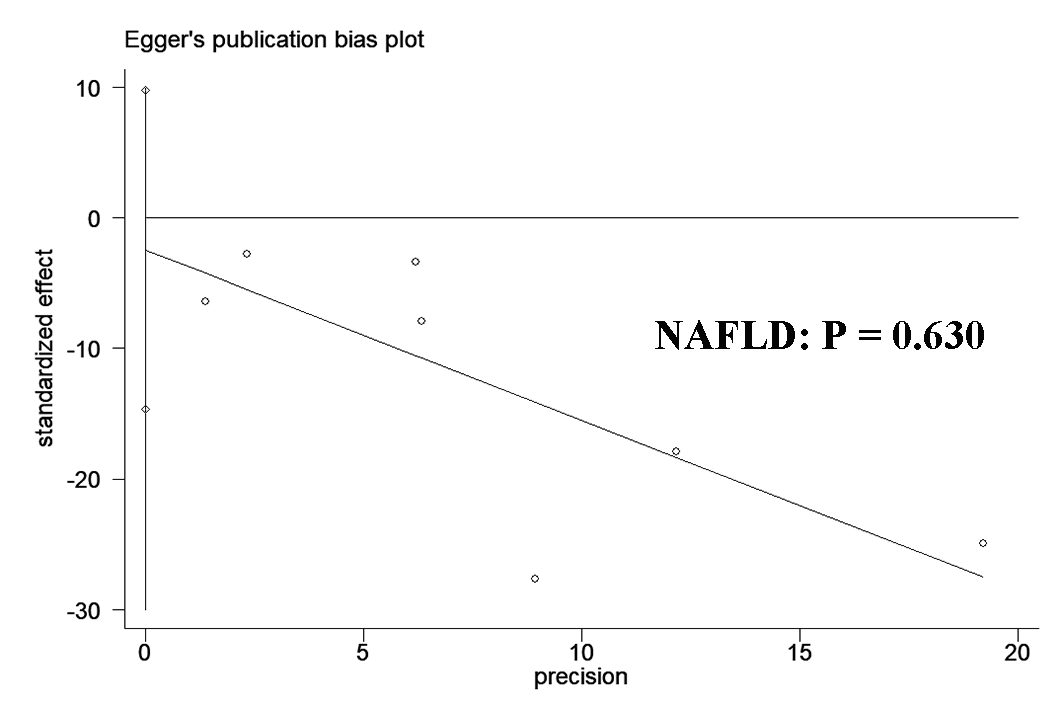


**Figure S1** Publication bias assessed by egger’s plot. (A) In the pooled analysis comparing the skeletal muscle index between nonalcoholic fatty liver disease (NAFLD) patients and normal people. NAFLD: nonalcoholic fatty liver disease.


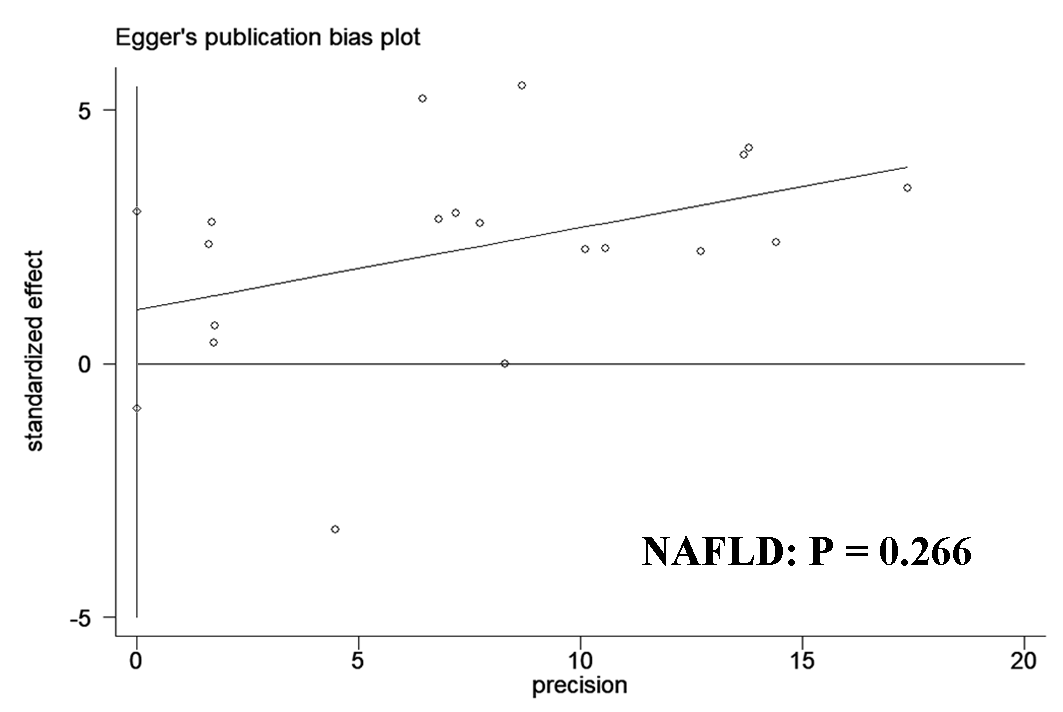


**Figure S2** Publication bias assessed by egger’s plot. (A) In the pooled analysis calculating the nonalcoholic fatty liver disease (NAFLD) incidence. NAFLD: nonalcoholic fatty liver disease.


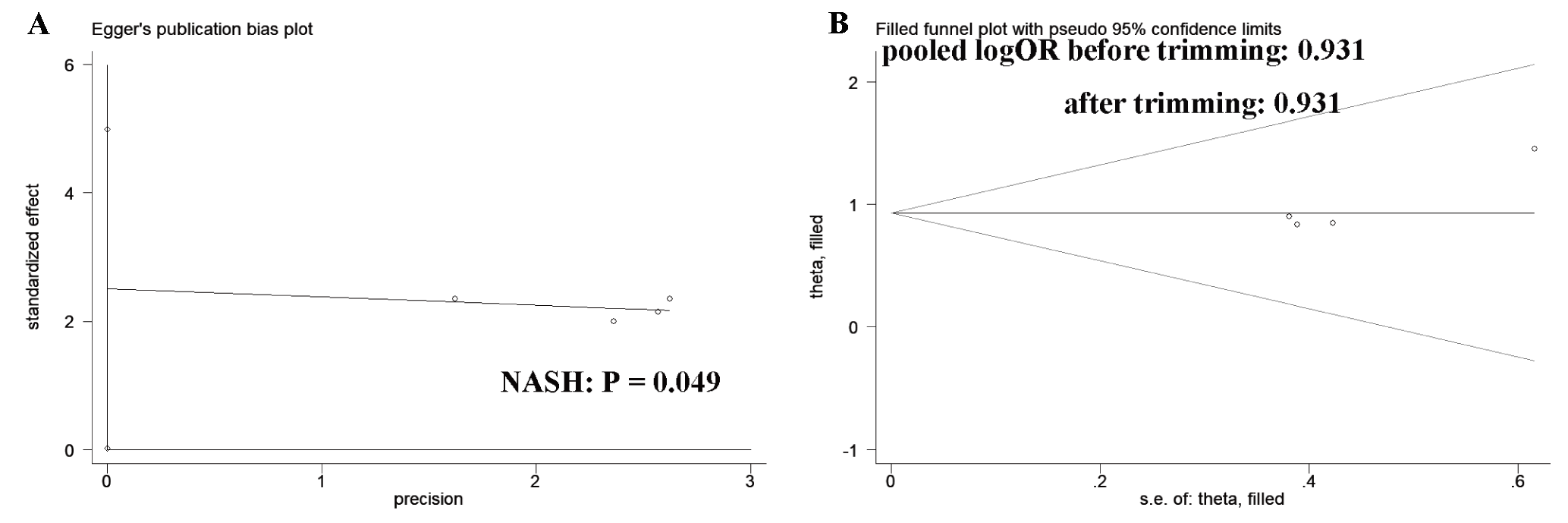


**Figure S3** (A) Publication bias assessed by egger’s plot in the pooled analysis calculating the nonalcoholic steatohepatitis (NASH) prevalence between nonalcoholic fatty liver disease (NAFLD) patients with and without sarcopenia. (B) Trim-and-fill analysis was conducted to assess the effect of publication bias on the interpretation of the results. NASH: non-alcoholic steatohepatitis; NAFLD: non-alcoholic fatty liver disease.


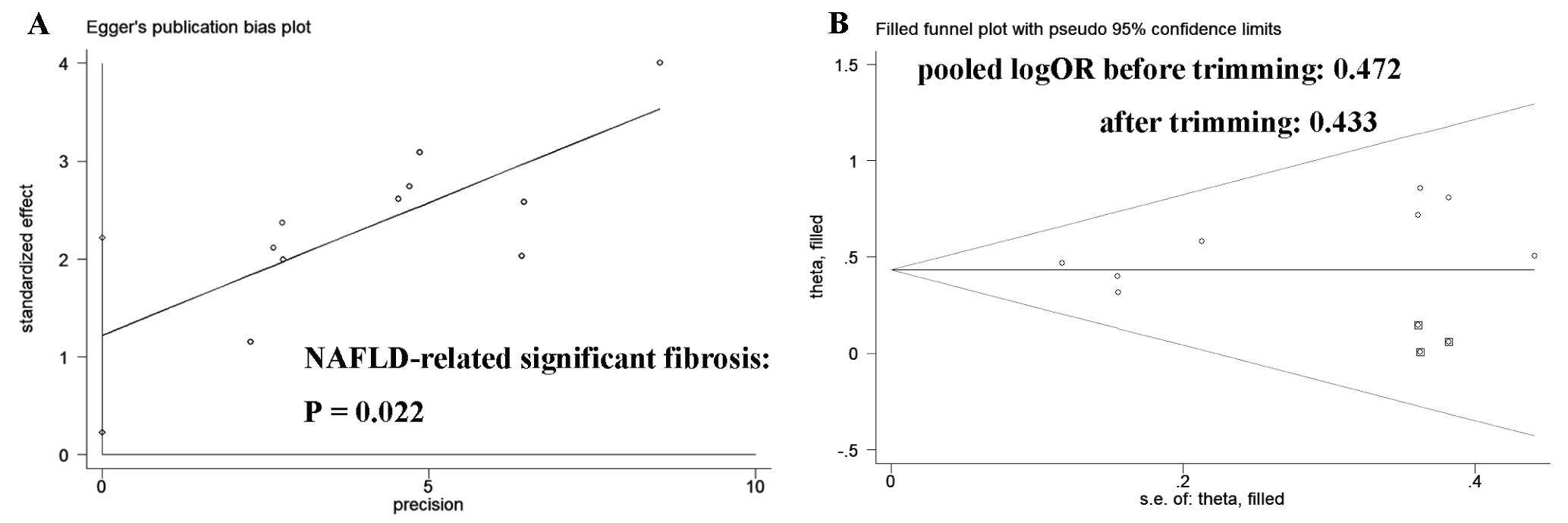


**Figure S4** (A) Publication bias assessed by egger’s plot in the pooled analysis calculating the nonalcoholic fatty liver disease (NAFLD) related advanced fibrosis incidence between NAFLD with and without sarcopenia. (B) Trim-and-fill analysis was conducted to assess the effect of publication bias on the interpretation of the results. NAFLD: nonalcoholic fatty liver disease.


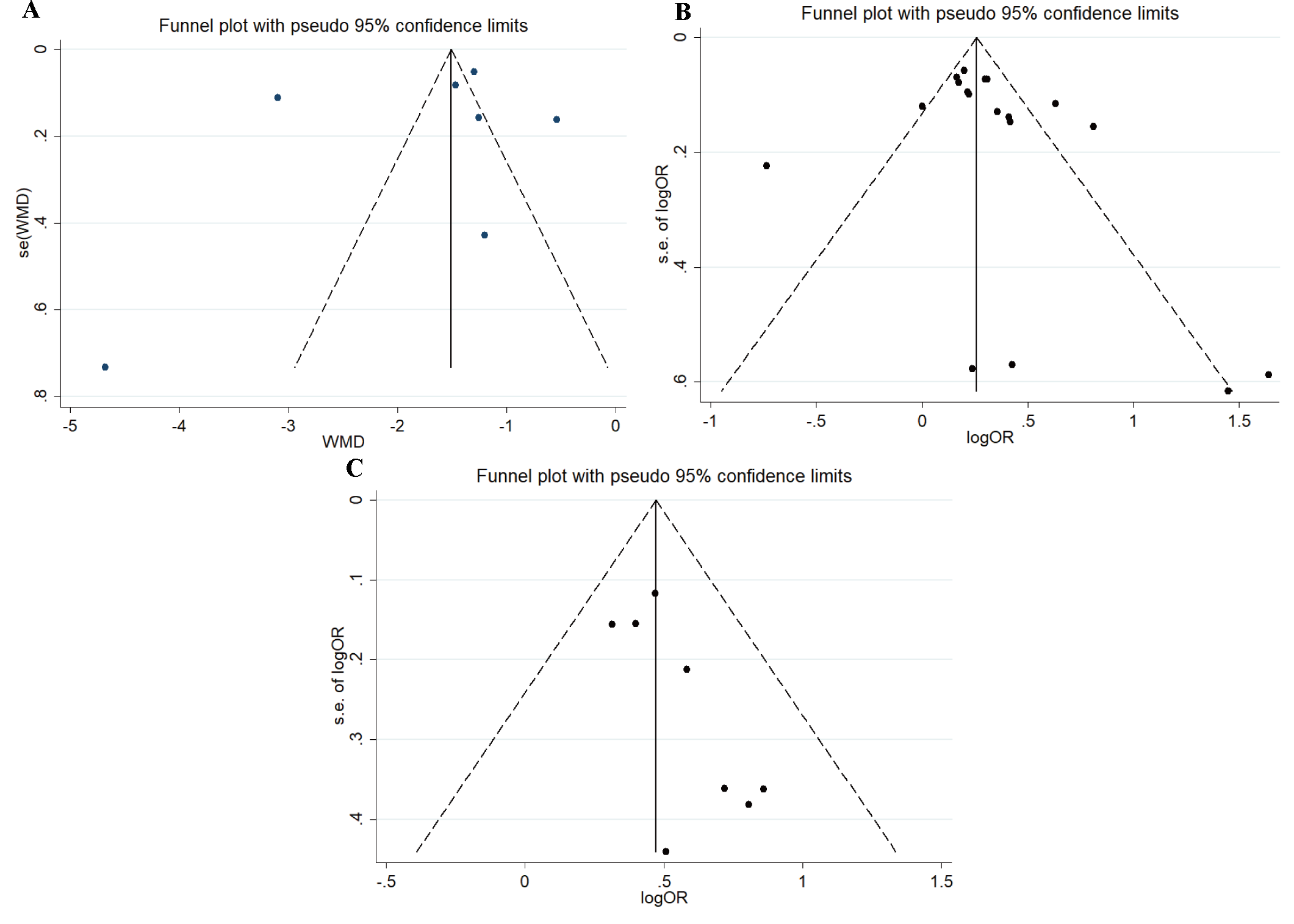
**Figure S5** Funnel plots. (A) In the pooled analysis comparing the skeletal muscle index between nonalcoholic fatty liver disease (NAFLD) patients and normal people; (B) In the pooled analysis calculating the NAFLD incidence between sarcopenia patients and normal people; (C) In the pooled analysis calculating the NAFLD - related advanced fibrosis incidence between NAFLD with and without sarcopenia. NAFLD: nonalcoholic fatty liver disease; OR: odds ratio; WMD: weighted mean difference.
